# The complete chloroplast genome of *Rhodobryum laxelimbatum* (Hampe ex Ochi) Z. Iwatsuki and T. J. Koponen

**DOI:** 10.1080/23802359.2021.1962759

**Published:** 2021-08-13

**Authors:** Shuangling Li, Fengjiao Shen, Shijia Zhang, Jingyuan Niu, Yulu Niu, Lin Li, Jiancheng Zhao

**Affiliations:** a College of Life Sciences, Hebei Normal University, Shijiazhuang, China; b School of Basic Medical Sciences, Xinxiang Medical University, Xinxiang, China; c College of Life Sciences, Hengshui University, Hengshui, China

**Keywords:** Bryales, Bryaceae, *Bryum*, Bryophytes, cardiovascular disease

## Abstract

*Rhodobryum laxelimbatum* (Bryophyta, Bryaceae) is one of the folk medicine resources in Southwest China, which has excellent potential for application in treating cardiovascular diseases. In this study, *R. laxelimbatum* was sequenced by high-throughput sequencing technology. The complete chloroplast genome is 124,632 bp in length with a quadripartite structure. Two inverted repeat regions are 9837 bp, separated by a large single copy region of 86,444 bp and a small single copy region of 18,514 bp. It encodes 118 unique genes, including 82 protein-coding genes, 32 tRNA genes, and four rRNA genes. The phylogenetic tree was constructed based on the complete chloroplast genome sequences of 18 bryophytes, downloaded from GenBank and acquired in this study. The phylogenetic analysis strongly indicated that *R. laxelimbatum* was the sister group of the clade which consists of *Mnium marginatum*, *Pohlia cruda* and *Pohlia nutans*. The *R. laxelimbatum* chloroplast genome sequence provides new genomic resources, which will improve its research, conservation, and application in the future.

The genus of *Rhodobryum* was established by German botanist Limpricht (1892) and was placed in the family of Bryaceae. *Rhodobryum* got the name because its leaves were clustered at the stem’s tip in a distinct rose-shaped pattern (*Rhodon* means rose, and *Bryum* is the type genus of Bryaceae). The Chinese name of *Rhodobryum*, ‘Da-ye-xian’, means mosses with big leaves because the leaves of *Rhodobryum* are larger than other Bryaceae species’ (Chen 1963). As a traditional folk medicine, the species of the genus *Rhodobryum* are broadly popular known to treat cardiovascular disease and nervous prostration (Li 1990; Hu et al. 2009, Li and Zhao 2009; Cai et al. 2011). Several pharmacological studies have attested to their efficacy (Harris 2008; Harris and Yang 2009), yet little is known about the chloroplast genome sequences of *Rhodobryum*. Therefore this study of *R. laxelimbatum* will help for better understanding of this genus and provide valuable information for the phylogeny of Bryaceae (Shi et al. 2021).

DNA of *Rhodobryum laxelimbatum* was extracted from dry leaves by a modified CTAB method (Li et al. 2013). The sample was collected from Haba Snow Mountain in Shangri-La, Yunnan, China (100.117°E, 27.313°N), and the voucher specimen (Shuo Shi, SZ5212; September 19, 2016) was deposited in the herbarium of Hebei Normal University, HBNU. The people in charge of the HBNU collection is Shuo Shi, email: shishuo@hebtu.edu.cn. With a paired-end (PE 150) genomic library acquired by the Illumina HiSeq X Ten platform (by Novogene Biotech Company, Beijing), the *R. laxelimbatum* complete chloroplast genome was assembled with Spades (Bankevich et al. 2012) and Geneious (Kearse et al. 2012), the boundaries among the repetitive and unique regions, we confirm such regions by mapping original reads (150 bp long) to the corresponding region. The sequence was annotated in DOGMA (Wyman et al. 2004) and edited in Sequin v15.50.

The complete chloroplast DNA sequence of *R. laxelimbatum* (GenBank accession No. MW147233) is 124,632 bp in length. Two inverted repeat regions (IRs) are 9837 bp long and separated by a large single copy (LSC) region of 86,444 bp and a small single copy (SSC) region of 18,514 bp. The genome contains 118 unique genes, including 82 protein-coding genes, 32 tRNA genes, and four rRNA genes (Detail information is available at GenBank: https://www.ncbi.nlm.nih.gov/nuccore/MW147233.1/#feature_MW147233.1). Through gene annotation, 15 genes (*trnL-GAU*, *trnA-UGC*, *ndhA*, *ndhB, rpl2*, *rpl16*, *petD*, *petB*, *trnV-UAC*, *trnL-UAA*, *trnK-UUU*, *trnG-GCC*, *atpF*, *rpoC1*, *ycf66*) contain one intron separately, and three genes (*clpP, ycf3 and rps12*) each possess two introns. The overall G/C content is 29.22%. Moreover, there are 158 simple sequence repeats (SSR) in the chloroplast genome. SSRs were detected by MISA (MicroSatellite identification tool) with the search parameters set at the minimum repeat threshold of mononucleotide 10 bp, dinucleotide 12 bp, trinucleotide, tetranucleotide and pentanucleotide 15 bp, and hexanucleotide 24 bp.

The ML tree was constructed with PhyloSuite v1.1.14 (Zhang et al. 2020) based on the complete chloroplast genomes of 17 mosses and one liverwort (*Marchantia polymorpha*, as outgroup). As we all know, there are majorly two types of chloroplast genome data deposited in the NCBI, contributing to the different orientations among the repetitive regions (IRa and IRb) and unique regions (LSC and SSC). Firstly, we used Mauve (Darling et al. 2004) to determine the gene collinearity of the chloroplast genome and manually adjust the specific structure of the genome. Then the whole genome data of 18 genomes were aligned by the MAFFT v7.222 (Katoh and Standley 2013) and manually adjusted with BioEdit v7.0.9.0 (Alzohairy 2011). Phylogenetic analysis was conducted based on maximum-likelihood analyses implemented in IQ-TREE (Nguyen et al. 2015) with a TVM + R3 + F model for 10,000 ultrafast (Minh et al. 2013) bootstraps, as well as the Shimodaira-Hasegawa-like approximate likelihood-ratio test (Guindon et al. 2010). The tree was edit in Figtree v1.4.3. In the tree (Figure 1), most clades were supported with high bootstrap values (100%) and *R. laxelimbatum* was the sister group of the clade which consists of *Mnium marginatum*, *Pohlia cruda* and *Pohlia nutans*.

**Figure 1.**
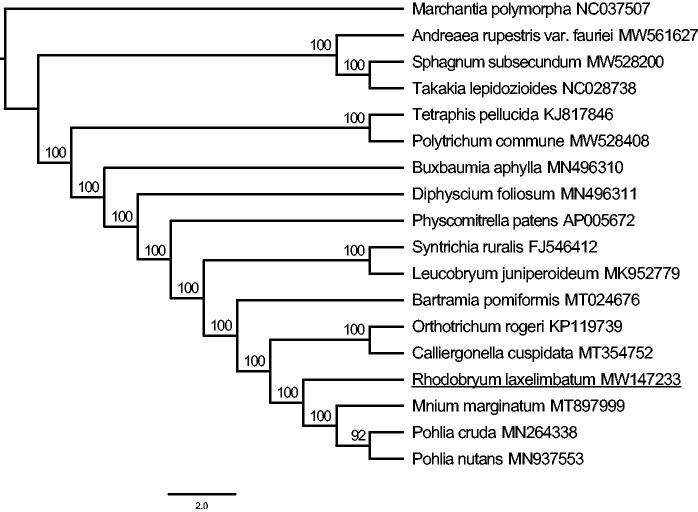
The ML tree was constructed based on 18 complete chloroplast genome sequences of bryophytes. Numbers on the branches were bootstrap values.

## Geolocation information

The study area of this research is in Yunnan, China.

## Data Availability

The genome sequence data that support the findings of this study are openly available in GenBank of NCBI at (https://www.ncbi.nlm.nih.gov/) under the accession number MW147233. The associated BioProject, SRA, and Bio-Sample number are PRJNA725408, SRR14332107 and SAMN18878621.
